# Seminal Interleukin-6 as a Biomarker of Inflammation, Oxidative Stress, and Sperm Dysfunction in Infertile Men

**DOI:** 10.3390/diseases14020049

**Published:** 2026-01-30

**Authors:** Loïc Koumba, Mariame Kabbour, Salma Ed-doumy, Mariem Norredine, Ahlam Zarhouti, Modou Mamoune Mbaye, Bouchra Ghazi, Noureddine Louanjli, Moncef Benkhalifa, Rajaa Ait Mhand, Ouafaa Aniq Filali

**Affiliations:** 1 Laboratory of Health, Environment and Biotechnology, Team of Physiopathology, Molecular Genetics and Biotechnology, Faculty of Sciences Ain Chock, Hassan II University of Casablanca, B.P 5366 Maarif, Casablanca 20100, Morocco; 2 Labomac IVF Centers and Clinical Laboratory Medicine, Casablanca 20100, Morocco; 3 IVF Center IRIFIV, Iris Clinic, Casablanca 20100, Morocco; 4 Laboratory of Chemistry-Physics and Biotechnology of Biomolecules and Materials, Faculty of Science and Technology Mohemmadia, Hassan II University of Casablanca, Casablanca 28806, Morocco; 5 Immunopathology-Immunotherapy-Immunomonitoring Laboratory, Faculty of Medicine, Mohammed VI University of Health and Sciences, Casablanca 82403, Morocco; 6 Faculty of Medicine, Mohammed VI University of Sciences and Health (UM6SS), Casablanca 82403, Morocco; 7 Mohamed VI Center for Research & Innovation (CM6RI), Rabat 10112, Morocco; 8 Reproductive Medicine, Developmental and Reproductive Biology, Regional University Hospital & School of Medicine and Peritox Laboratory, Picardie University Jules Verne, 80054 Amiens, France

**Keywords:** male infertility, seminal IL-6, sperm quality, oxidative stress, leukocytospermia, bacteriospermia, sperm DNA fragmentation, sperm chromatin decondensation

## Abstract

Background/Objectives: Interleukin-6 (IL-6), a pleiotropic cytokine involved in immune regulation, is consistently detected in human semen, even in the absence of overt infection. Its contribution to sperm dysfunction, oxidative stress, and inflammation remains incompletely understood. This study evaluated the associations between seminal IL-6 concentrations and markers of semen quality, oxidative stress, nuclear integrity, and genital tract inflammation in infertile men. Methods: A cohort of 204 infertile men was assessed. Seminal IL-6 was quantified by electrochemiluminescence immunoassay. Semen parameters, malondialdehyde (MDA), catalase (CAT) activity, sperm DNA fragmentation index (DFI), sperm chromatin decondensation index (SDI), leukocytospermia, and bacteriospermia were measured. Analyses included correlation testing, IL-6 threshold stratification (<30, 30–60, 60–100, ≥100 pg/mL), and multivariate regression. Results: IL-6 was detectable in all samples (median: 31.52 pg/mL; range: 1.5–5000 pg/mL). Higher IL-6 levels were significantly associated with reduced sperm concentration, progressive motility, and vitality, and with increased DFI, SDI, MDA, leukocyte counts, and bacteriospermia (*p* < 0.001). In multivariate models, IL-6 independently predicted reduced progressive motility (β = −0.005; *p* = 0.032) and elevated leukocyte count (β = 0.0018; *p* < 0.0001). Logistic regression further showed that IL-6 increased the odds of DFI ≥ 30%, SDI ≥ 30%, and bacteriospermia (*p* < 0.05). Conclusions: Seminal IL-6 emerges as a sensitive biomarker of immuno-oxidative stress and sperm dysfunction in infertile men. Its integration into clinical evaluation may improve the assessment of inflammatory and oxidative contributors to male infertility.

## 1. Introduction

Infertility is defined as the inability of a couple to conceive after 12 months of regular, unprotected intercourse and affects approximately 15% of reproductive-age couples worldwide [1,2]. A purely male factor is identified in 20–30% of cases, and male involvement is present in nearly half, either as an isolated or combined contributor [3,4,5]. Among male etiologies, inflammation of the genital tract is recognized as a major determinant.

This inflammatory process involves a complex network of cytokines and immunomodulatory mediators capable of disrupting spermatogenesis, altering the testicular microenvironment, and impairing sperm function, including motility, acrosome reaction, and DNA integrity [5,6,7,8,9]. Among these mediators, interleukin-6 (IL-6) plays a central regulatory role in inflammatory and immune responses. Unlike TNF-α or IL-1β, which are primarily implicated in acute-phase reactions, or IL-8, which governs neutrophil recruitment, IL-6 contributes to both the initiation and the chronic maintenance of inflammation, thereby bridging acute and sustained immune activation [6,10,11,12,13].

Multiple studies have reported the frequent detection of IL-6 in human semen, even in the absence of clinical signs of infection [12,14,15]. However, the evidence remains highly variable due to differences in study populations, methodological approaches, and diagnostic thresholds, which range widely from about 15 pg/mL [16] to more than 200 pg/mL [17]. Although several case–control studies comparing fertile and infertile men have reported higher seminal IL-6 levels in infertile individuals [18,19,20,21], these findings remain heterogeneous and rarely explore the internal biological gradients that may exist within infertile populations. Such gradients could provide important insight into disease severity, prognostic stratification, and potential therapeutic monitoring.

To address these gaps, the present study adopts an intra-cohort analytical design in a homogeneous population of infertile men to characterize dose–response relationships between seminal IL-6 concentrations and key markers of sperm dysfunction. Specifically, this approach aims to: (i) identify clinically meaningful IL-6 thresholds associated with progressive impairment of standard semen parameters (concentration, progressive motility, vitality), inflammatory markers (leukocytospermia, bacteriospermia), and oxidative/nuclear damage indicators (MDA, catalase, DFI, SDI); and (ii) compare the predictive performance of IL-6 with that of conventional biomarkers within the inflammatory–oxidative spectrum observed in infertile men.

## 2. Materials and Methods

### 2.1. Study Population

A total of 204 infertile men aged 24–56 years who consulted the LABOMAC laboratory (Casablanca, Morocco) for semen analysis were enrolled in this study.

### 2.2. Inclusion and Exclusion Criteria

Inclusion criteria were the absence of antibiotic treatment within the previous three months, provision of a complete ejaculate, and signed informed consent. Exclusion criteria included azoospermia, cryptozoospermia, documented genital tract infection, chronic illness, or a recent febrile episode.

### 2.3. Semen Collection

Participants observed 3–5 days of sexual abstinence prior to sample collection. To minimize contamination, they were instructed to void urine, wash their hands and genital area with antimicrobial soap, and rinse with sterile saline solution. Semen samples were obtained by masturbation in a dedicated laboratory room and collected in sterile, non-cytotoxic containers.

### 2.4. Semen Analysis

Semen analyses were performed according to World Health Organization guidelines [2]. After liquefaction at 37 °C for 30 min, sperm concentration and motility were assessed using computer-assisted semen analysis (CASA; Spermolyzer^TM^, Mira Lab, Cairo, Egypt).

Sperm morphology was evaluated on Schorr’s hematoxylin-stained smears according to the modified David classification. Vitality was assessed using 2% eosin staining, with a minimum of 200 cells manually counted.

Leukocyte concentration was determined using either peroxidase staining or CD45 immunolabeling, depending on reagent availability at the time of analysis. Systematic bacteriological cultures were performed on selective media to identify urogenital pathogens.

### 2.5. Definition of Leukocytospermia

Leukocytospermia was defined as ≥1 × 10^6^ leukocytes/mL [2] and assessed using the LeucoScreen^®^ test (Fertipro, Beernem, Belgium). Briefly, 10 µL of semen was mixed with 10 µL of a working solution prepared by diluting 30 µL of 3% hydrogen peroxide in LeucoScreen reagent to a final volume of 1 mL. After 2 min of incubation, preparations were examined under light microscopy (×40 objective). Leukocytes, identified by their brown coloration, were manually counted and expressed as cells per milliliter.

### 2.6. Bacteriological Analysis

Semen aliquots were diluted 1:10 (*v*/*v*) in sterile isotonic saline (0.15 M NaCl) and plated on enriched chocolate agar (Polyvitex^®^, bioMérieux, Marcy-l’Étoile, France). Cultures were incubated at 37 °C under 5% CO_2_ for 24–72 h.

Bacteria were identified using biochemical methods and automated systems (Vitek 2 Compact^®^ and API 10S^®^, bioMérieux, France). Pathogenicity thresholds were interpreted in accordance with the French *Référentiel en Microbiologie Médicale* [22], and only bacterial species exceeding their species-specific pathogenicity thresholds were included in the analysis. Commensal flora and non-pathogenic isolates were excluded.

### 2.7. Sperm DNA Fragmentation and Chromatin Decondensation

#### 2.7.1. Sample Preparation

All semen samples were processed within one hour after collection. After centrifugation (1000× *g*, 15 min), seminal plasma was stored at −20 °C for biochemical analyses. The sperm pellet was washed in phosphate-buffered saline (PBS, pH 7.4), centrifuged (400× *g*, 10 min), and smeared onto two separate slides: one for the TUNEL assay (DNA fragmentation) and another for aniline blue staining (chromatin condensation).

Slides were air-dried, fixed in PBS–formaldehyde (90/10, *v*/*v*) at 37 °C for 30 min, rinsed, and permeabilized for 1 min in a solution containing distilled water (98%), sodium citrate (1%), and Triton X-100 (1%).

#### 2.7.2. DNA Fragmentation Index (DFI)

DNA fragmentation was quantified using the TUNEL assay (In Situ Cell Death Detection Fluorescein Kit, Roche Diagnostics GmbH, Mannheim, Germany), strictly following the manufacturer’s instructions. Slides were analyzed under a fluorescence microscope (Nikon Eclipse 80i, Nikon Corporation, Tokyo, Japan) using a ×100 oil-immersion objective. For each sample, a minimum of 200 spermatozoa were evaluated. Fluorescent nuclei were classified as fragmented, and the DFI was calculated as the percentage of TUNEL-positive spermatozoa.

Consistent with established thresholds, a DFI ≥ 30% was considered abnormal and indicative of impaired sperm nuclear integrity [23].

#### 2.7.3. Sperm Chromatin Decondensation Index (SDI)

Chromatin condensation was assessed using aniline blue staining, according to the protocol described by Belloc et al. [24]. Slides were stained for 15 min, rinsed, air-dried, and examined by bright-field microscopy (×100 objective). For each sample, 200 spermatozoa were counted.

Spermatozoa exhibiting dark-blue staining, reflecting excessive histone retention, were classified as abnormal. An SDI ≥ 30% was considered pathological.

### 2.8. Seminal IL-6 Measurement

Interleukin-6 concentrations were quantified in seminal plasma using a sandwich electrochemiluminescence immunoassay (ECLIA; Elecsys^®^ IL-6 kit, Roche Diagnostics GmbH, Mannheim, Germany) following the manufacturer’s recommendations.

The analytical range was 1.5–5000 pg/mL, enabling sensitive detection of subclinical inflammation and accurate measurement under moderate to severe inflammatory conditions. To account for the heterogeneity of thresholds reported in the literature (≈15–200 pg/mL), seminal IL-6 concentrations were further categorized into four groups: <30, 30–60, 60–100, and ≥100 pg/mL. These cut-off values were derived from the empirical distribution of IL-6 in our cohort, approximately corresponding to the 25th, 50th, and 75th percentiles and rounded to the nearest integer to facilitate clinical interpretation. Because of the right-skewed distribution of IL-6, the resulting groups are not numerically equivalent in size but reflect the underlying biological heterogeneity of the population.

### 2.9. Catalase Activity

Catalase activity was measured spectrophotometrically according to the method of Aebi [25], by monitoring hydrogen peroxide (H_2_O_2_) degradation at 240 nm. The reaction mixture (1 mL) contained 7.5 mM H_2_O_2_ in 50 mM potassium phosphate buffer (pH 7.4), with 10 µL of seminal plasma added. Catalase activity was calculated using the molar extinction coefficient of H_2_O_2_ (ε = 0.0394 mM^−1^·cm^−1^) and expressed as µmol of H_2_O_2_ degraded per minute per milligram of protein.

### 2.10. Malondialdehyde (MDA) Measurement

Lipid peroxidation was assessed using the thiobarbituric acid reactive substances (TBARS) assay [26]. Briefly, 100 µL of seminal plasma was mixed with 900 µL of a solution containing 0.375% thiobarbiturbituric acid (TBA), 15% trichloroacetic acid (TCA), and 0.25 M hydrochloric acid (HCl). Samples were incubated at 100 °C for 20 min, cooled on ice, and centrifuged at 1000× *g* for 10 min at 4 °C. Absorbance of the supernatant was measured at 535 nm. MDA concentration was calculated using an extinction coefficient of 1.56 × 10^5^ M^−1^·cm^−1^ and expressed as nmol per mg of protein.

### 2.11. Statistical Analysis

Statistical analyses were performed using GraphPad Prism (version 10.4.1, GraphPad Software, Boston, MA, USA). As the data did not follow a normal distribution (Kolmogorov–Smirnov test), results are presented as medians with interquartile ranges (IQR).

Comparisons of semen quality, inflammatory markers, and oxidative stress parameters across IL-6 groups were performed using the Kruskal–Wallis test, followed by Dunn’s post hoc test with Bonferroni correction when appropriate. Spearman’s rank coefficient (ρ) was used to assess correlations.

Multivariate regression models (linear or logistic, depending on the outcome variable) were applied to identify independent associations between IL-6 levels and biological parameters. Age, smoking status, and alcohol consumption were systematically included as covariates in all regression models to account for their potential confounding effects. Results are expressed as β coefficients (linear regression) or odds ratios (OR, logistic regression), with 95% confidence intervals (CI).

To evaluate the diagnostic performance of seminal IL-6, receiver operating characteristic (ROC) analyses were conducted for the prediction of (i) DFI ≥ 30%, (ii) SDI ≥ 30%, and (iii) bacteriospermia. For comparison, ROC analyses were also performed for leukocyte concentration, malondialdehyde (MDA), and catalase (CAT). For each marker and outcome, the area under the curve (AUC), optimal cut-off value (Youden index), sensitivity, and specificity were calculated. Only the numerical results of these ROC analyses are reported in the manuscript; ROC curve plots were not included.

A *p*-value < 0.05 was considered statistically significant.

### 2.12. Quality Control

All semen analyses and biochemical assays were performed in duplicate, and results were independently validated by two blinded analysts. In case of discrepancy, a senior biologist reviewed the data or repeated the assay to ensure reliability.

### 2.13. Ethical Considerations

The study protocol was approved by the Biomedical Research Ethics Committee of UM6SS (reference: CE/UM6SS/09/23; 25 July 2023). Written informed consent was obtained from all participants prior to inclusion. All data were anonymized and analyzed in accordance with the principles of the Declaration of Helsinki (2013 revision).

## 3. Results

### 3.1. Description of the Study Population

**Figure 1:**
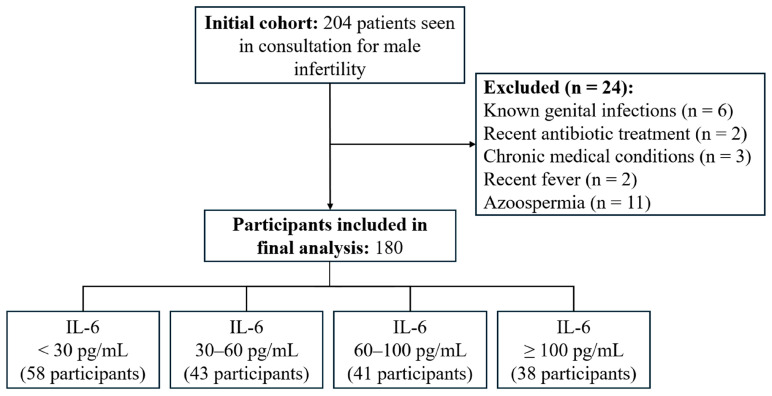
Flowchart of participant selection for the study.

Of the 204 patients initially recruited, 24 were excluded due to non-eligibility (Figure 1). The final analysis included 180 participants. IL-6 was detectable in all seminal plasma samples, with a median concentration of 31.52 pg/mL (Q1 = 12.39 pg/mL; Q3 = 70.11 pg/mL; IQR = 57.72 pg/mL; range: 1.5–5000 pg/mL).

To investigate potential dose–response relationships, participants were stratified into four IL-6 concentration groups (<30, 30–60, 60–100, and ≥100 pg/mL). These categories reflect the empirical distribution of IL-6 in the cohort and correspond approximately to the 25th, 50th, and 75th percentiles, rounded to the nearest integer to facilitate clinical interpretation.

### 3.2. Sociodemographic Characteristics

Table 1 summarizes the distribution of age, smoking status, and alcohol consumption across IL-6 groups. The mean age of participants was 36.06 ± 8.01 years. Among them, 37 (20.6%) reported smoking and 25 (13.9%) reported regular alcohol consumption. No statistically significant differences were observed between IL-6 groups for any sociodemographic or behavioral variable.

### 3.3. Semen Parameters, Inflammatory and Oxidative Markers

**Figure 2:**
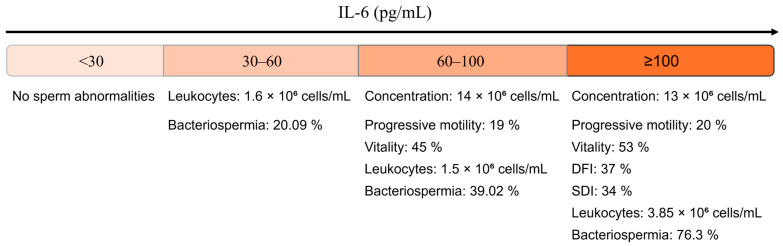
Stratification of seminal IL-6 concentrations and corresponding profiles of key semen parameters.

Figure 2 provides a descriptive overview of seminal characteristics across the four IL-6 concentration groups, using WHO reference thresholds for classifying semen parameters [2]. In the <30 pg/mL group, no semen abnormalities were detected according to WHO criteria. In the 30–60 pg/mL group, leukocytospermia (1.6 × 10^6^ cells/mL) and bacteriospermia (20.09%) were observed.

From 60 to 100 pg/mL, alterations included reduced sperm concentration, progressive motility, and vitality, together with increased leukocyte counts and a higher proportion of bacteriospermia (39.02%). The ≥100 pg/mL group exhibited the most marked abnormalities, including low semen parameters, elevated DFI (37%) and SDI (34%), leukocytospermia (3.85 × 10^6^ cells/mL), and the highest rate of bacteriospermia (76.3%).

This figure provides a descriptive summary without statistical inference and visually complements the analytical findings reported later in Section 3.

Semen quality varied significantly across IL-6 groups (Table 2). Participants with IL-6 concentrations < 30 pg/mL exhibited the most favorable seminal profile, with a median sperm concentration of 35 × 10^6^/mL, progressive motility of 45.4%, and vitality of 70%. These parameters declined progressively with increasing IL-6 levels, reaching their lowest values in the ≥100 pg/mL group (*p* < 0.001). No significant differences were observed for normal sperm morphology (*p* = 0.378).

Conversely, DFI, SDI, leukocyte counts, bacteriospermia, and MDA concentrations increased with rising IL-6 levels, with significant differences emerging from the 60–100 pg/mL group. Catalase activity did not differ significantly between groups (*p* = 0.326).

Post hoc analyses (Dunn’s test with Bonferroni correction) confirmed that IL-6 ≥ 60 pg/mL was significantly associated with reduced sperm concentration, progressive motility, and vitality, as well as increased DFI, SDI, leukocytospermia, and bacteriospermia compared with the <30 pg/mL group (*p* < 0.05). These differences were most pronounced in the ≥100 pg/mL group (*p* ≤ 0.0086). MDA levels were significantly elevated only in the ≥100 pg/mL group (*p* = 0.0325).

**Figure 3:**
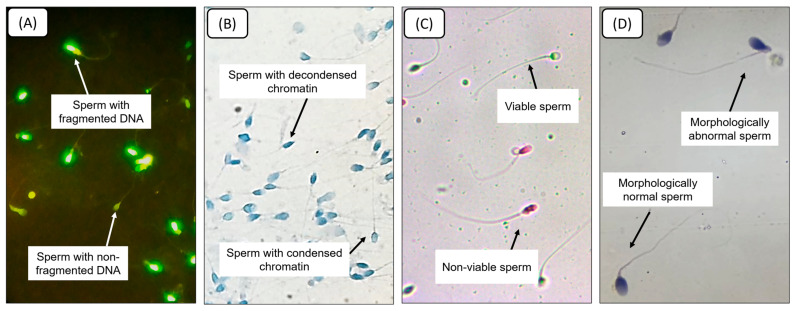
Representative microscopic images of sperm analyses: (**A**) TUNEL assay identifying spermatozoa with fragmented DNA; (**B**) aniline blue staining revealing chromatin decondensation; (**C**) eosin vitality test distinguishing live from dead spermatozoa; (**D**) Schorr’s hematoxylin staining for sperm morphology assessment.

To illustrate the microscopic alterations underlying the quantitative measurements, Figure 3 presents representative images of spermatozoa with fragmented versus non-fragmented DNA (TUNEL assay), condensed versus decondensed chromatin (aniline blue staining), live versus dead cells (eosin vitality test), and morphologically normal versus abnormal forms (H&E staining). These examples visually complement the quantitative data by showing the corresponding structural and functional changes.

### 3.4. Correlation Analyses

**Figure 4:**
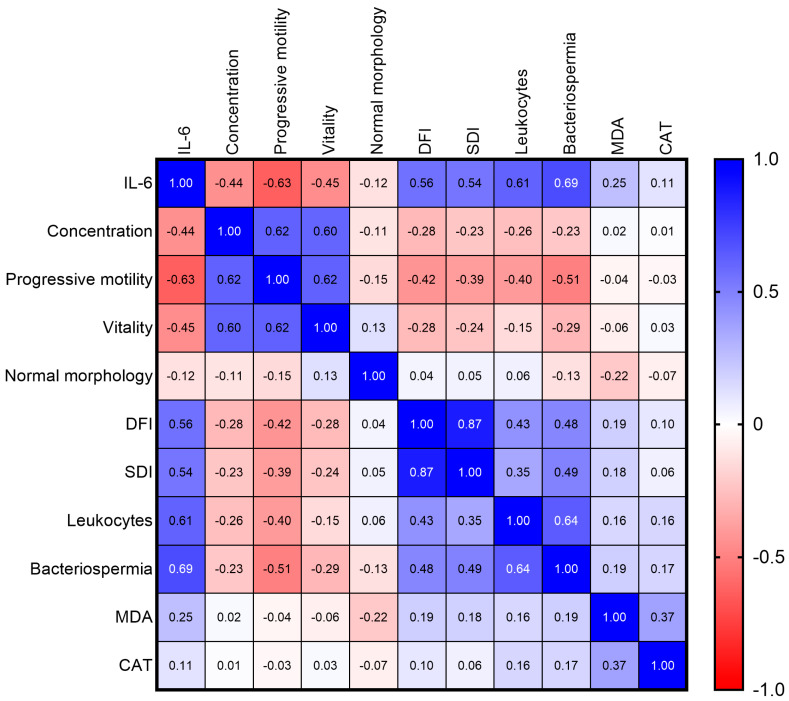
Spearman correlation matrix of seminal, inflammatory, oxidative, and nuclear parameters. Correlation coefficients (ρ) are represented on a color scale, with positive values shown in red and negative values in blue. Stronger color intensity indicates higher absolute correlation values. Statistically significant correlations (*p* < 0.05) appear with enhanced contrast.

To provide an integrated overview of the interrelationships among the seminal parameters investigated, a Spearman correlation matrix was generated (Figure 4). This visualization highlights several strong associations within the cohort.

IL-6 exhibited the most pronounced correlations, showing strong positive associations with bacteriospermia (ρ = 0.694), leukocyte concentration (ρ = 0.614), DFI (ρ = 0.560), and SDI (ρ = 0.543). In contrast, the strongest negative correlations were observed with progressive motility (ρ = −0.628), vitality (ρ = −0.452), and sperm concentration (ρ = −0.443).

Beyond the central role of IL-6, the matrix revealed additional biologically relevant relationships, including a very strong correlation between DFI and SDI (ρ = 0.867), as well as inverse associations between classical semen parameters (concentration, motility, vitality) and markers of inflammation or nuclear integrity.

### 3.5. Bacterial Profile

**Figure 5:**
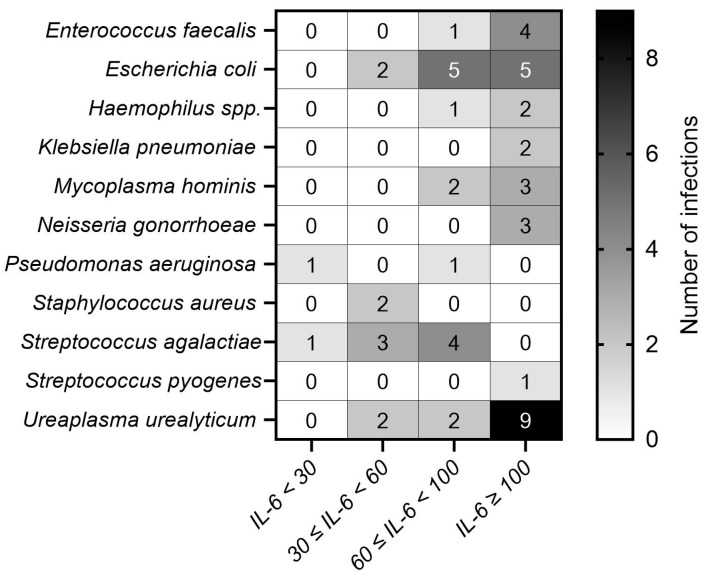
Distribution of bacterial species according to IL-6 groups.

Bacterial infection was identified in 56 cases (31.1%), with 11 distinct species isolated (Figure 5). Each infected sample harbored only one bacterial species. In accordance with the microbiological criteria used in the study, only organisms exceeding their species-specific pathogenicity thresholds were considered; commensal flora and non-pathogenic isolates were systematically excluded from the analysis.

Bacterial diversity increased progressively with IL-6 concentration. In the <30 pg/mL group, only Pseudomonas aeruginosa and Streptococcus agalactiae were detected. In the 30–60 pg/mL group, isolates included *Escherichia coli* (n = 2), *Staphylococcus aureus* (n = 2), *Ureaplasma urealyticum* (n = 2), and *S. agalactiae* (n = 3). The 60–100 pg/mL group showed 16 infections, predominantly *E. coli* (n = 5) and *S. agalactiae* (n = 4). In the ≥100 pg/mL group, 29 infections were recorded, with *U. urealyticum* (n = 9) and *E. coli* (n = 5) being most frequent, followed by *Enterococcus faecalis* (n = 4), *Mycoplasma hominis* (n = 3), and *Neisseria gonorrhoeae* (n = 3).

### 3.6. Diagnostic Performance of IL-6 and Related Biomarkers

To assess the potential value of seminal IL-6 as a biomarker of sperm impairment, ROC analyses were performed for the prediction of DFI ≥ 30%, SDI ≥ 30%, and bacteriospermia. For comparison, ROC analyses were also conducted for leukocyte concentration, MDA, and catalase (CAT).

As summarized in Table 3, IL-6 exhibited good to excellent diagnostic performance for all three outcomes, with AUC values of 0.859 and 0.855 for DFI ≥ 30% and SDI ≥ 30%, respectively, and 0.927 for bacteriospermia. Leukocyte concentration also showed high performance, particularly for bacteriospermia (AUC = 0.904), whereas MDA and CAT displayed more modest diagnostic accuracies.

### 3.7. Multivariate Analyses

Multivariate linear regression, adjusted for age, smoking status, and alcohol consumption, identified IL-6 as an independent predictor of reduced progressive motility (β = −0.005; *p* = 0.032) and elevated leukocyte count (β = 0.0018; *p* < 0.0001). After adjustment for these covariates, no independently significant associations were found between IL-6 and sperm concentration, vitality, morphology, MDA, or catalase activity (Table 4).

Logistic regression analysis, likewise adjusted for age, smoking status, and alcohol consumption, showed that IL-6 significantly increased the odds of DFI ≥ 30% (OR = 1.0007; *p* = 0.032), SDI ≥ 30% (OR = 1.0006; *p* = 0.027), and bacteriospermia (OR = 1.0325; *p* < 0.001).

## 4. Discussion

This study examined the relationship between seminal IL-6 concentrations and key indicators of sperm dysfunction, oxidative stress, and inflammation in infertile men. IL-6 was detected in all samples, confirming its near-ubiquitous presence in the male reproductive tract [27,28]. Concentrations varied markedly (median: 31.52 pg/mL; IQR: 57.72 pg/mL), reflecting substantial interindividual variability in inflammatory activation, a finding consistent with evidence that IL-6 participates in the immunoregulatory milieu of the male genital tract and may reflect subclinical inflammation, asymptomatic infection, or environmental exposures [13,29].

This work provides three novel and complementary contributions to the understanding of seminal inflammation in male infertility. First, we established four clinically interpretable IL-6 thresholds (<30, 30–60, 60–100, and ≥100 pg/mL), revealing a coherent dose–response gradient across functional, inflammatory, and oxidative parameters. Second, IL-6 emerged as an independent predictor of nuclear instability (DFI, SDI ≥ 30%) and bacteriospermia, even after adjustment for age, smoking, and alcohol consumption. Third, IL-6 demonstrated superior diagnostic performance compared with leukocyte count, MDA, and catalase for predicting bacteriospermia, DFI ≥ 30%, and SDI ≥ 30%, reinforcing its relevance as a quantitative biomarker of seminal immuno-oxidative stress.

Unlike case–control studies that rely on a binary “normal vs. elevated” interpretation, an intra-cohort analytical design enables the characterization of continuous biological associations within infertile men. While this approach does not replace fertile–infertile comparisons nor establish physiological reference values, it provides complementary insights into internal dose–response patterns and potential risk stratification. This design rationale is essential to understand the added value of identifying IL-6 gradients within a clinically homogeneous infertile population.

A key methodological strength is the intra-cohort analytical design. Unlike case–control studies contrasting fertile and infertile populations, this approach characterizes continuous biological associations within infertile men and identifies clinically meaningful internal gradients. Although this design does not establish physiological reference values, it provides complementary insight into dose-dependent inflammatory and oxidative alterations. The IL-6 stratification approach revealed consistent patterns across semen quality, inflammation, and oxidative stress. Alterations appeared above 30 pg/mL, including reduced progressive motility, leukocytospermia (1.60 × 10^6^ cells/mL), and bacteriospermia in approximately 20% of participants. Above 60 pg/mL, abnormalities intensified, with reduced vitality and higher DFI/SDI, while concentrations ≥ 100 pg/mL were associated with the most severe impairments and significantly elevated MDA, indicating established oxidative injury. However, although IL-6 levels were significantly associated with nuclear damage markers (DFI, SDI), the present study does not provide direct experimental evidence demonstrating that IL-6–induced oxidative stress is causally responsible for these alterations. These associations should therefore be interpreted as reflective of a broader inflammatory–oxidative environment rather than proof of mechanistic causation.

**Figure 6:**
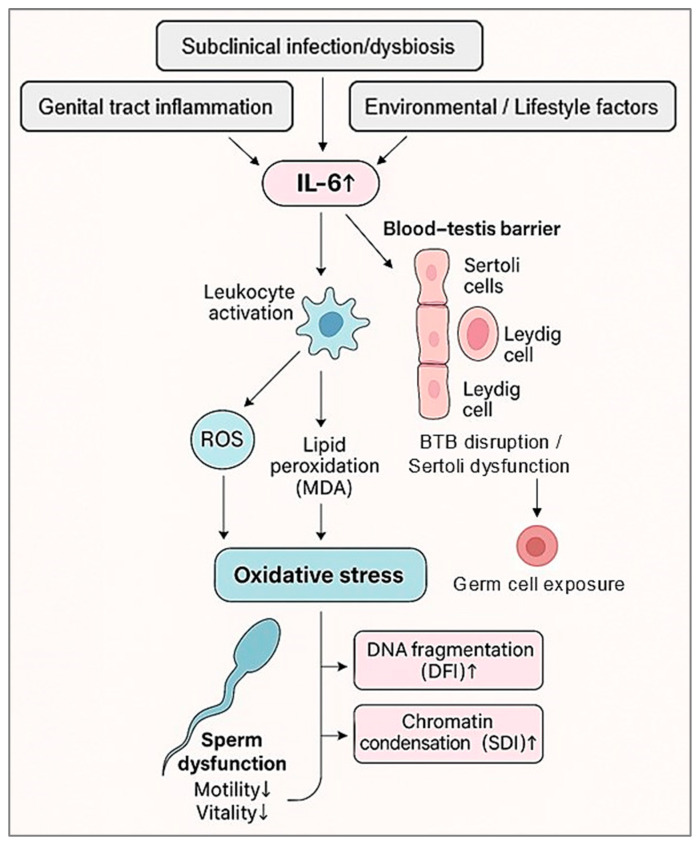
Conceptual and hypothetical model illustrating the potential interactions be-tween seminal interleukin-6 (IL-6), inflammatory activity, oxidative stress, microbial imbalance, and sperm functional and nuclear alterations in infertile men. This schematic representation is not derived from direct mechanistic experimentation in the present study but integrates the statistically observed associations with previously published biological evidence. Elevated seminal IL-6 levels are associated with increased leukocytospermia, bacteriospermia, and markers of oxidative stress, including lipid peroxidation. These inflammatory and oxidative conditions may coexist with impaired sperm motility, vitality, and nuclear integrity, reflected by increased DNA fragmentation index (DFI) and sperm chromatin decondensation index (SDI). The arrows represent potential biological interactions rather than confirmed causal pathways. This figure is intended to provide an integrative conceptual framework to support interpretation of the observed associations.

Several biological mechanisms described in previous studies may contextualize these findings. IL-6 can compromise Sertoli cell function by disrupting the blood–testis barrier (BTB) via MAPK/ERK signaling, altering tight-junction proteins such as occludin and claudins, and impairing junctional turnover [30]. These changes may expose developing germ cells to inflammatory and oxidative mediators. IL-6 may also impair Leydig cell steroidogenesis by downregulating key enzymes (CYP11A1, 3β-HSD, StAR), reducing intratesticular testosterone and weakening endocrine support for spermatogenesis [31]. Furthermore, IL-6–driven inflammation promotes excess ROS production through leukocyte activation and mitochondrial dysregulation, contributing to lipid peroxidation (MDA) and nuclear instability (DFI, SDI). Taken together, these mechanistic insights provide a coherent interpretative framework for understanding how IL-6–driven inflammatory and oxidative processes may converge to affect sperm function and nuclear integrity. To synthesize these interactions, Figure 6 presents a conceptual model integrating our observed associations with established biological pathways.

Oxidative imbalance emerged as a central feature of this IL-6 gradient. IL-6 was positively correlated with MDA but showed no significant association with catalase activity, suggesting disproportionate ROS generation relative to antioxidant capacity. This profile is consistent with prior studies linking IL-6 to oxidative stress and impaired sperm function [29]. In the <30 pg/mL group, both MDA (423.3 nmol/mg) and catalase (467.8 µmol/min/mg) remained relatively elevated, an arrangement compatible with an early compensated redox state. As IL-6 concentrations increased, MDA rose markedly while catalase did not increase proportionally, indicating a shift toward net oxidative imbalance accompanied by declining motility and vitality and increasing DFI and SDI, in accordance with evidence on ROS-mediated sperm damage [32,33,34,35].

The inflammatory component was reinforced by the strong association between IL-6 and leukocytospermia (ρ = 0.64). Leukocyte concentration was directly quantified in all samples using the WHO-recommended peroxidase test, confirming the presence and/or magnitude of inflammatory cell recruitment within the seminal milieu. Leukocytes are major sources of cytokines and ROS and may sustain an IL-6–dependent inflammatory loop [3,36]. Building on this inflammatory profile, the concomitant increase in bacteriospermia and leukocytospermia suggests a potential interaction between microbial colonization/infection and inflammatory activation within the male genital tract. While bacterial presence can initiate leukocyte recruitment, inflammation-induced alterations of the seminal microenvironment may, in turn, promote dysbiosis and facilitate microbial persistence. However, the causal direction cannot be established in a cross-sectional observational design [5,37]. In continuity with this pattern, although no specific microorganism corresponded to any particular IL-6 category, higher IL-6 levels coincided with greater microbial diversity, consistent with broad immune activation through PRR–NF-κB or MAPK pathways [38,39]. The <30 pg/mL group represents the lowest inflammatory burden with-in infertile men and should not be considered a physiological control.

Multivariate analyses further strengthened the independent role of IL-6 in seminal dysfunction. After adjustment for age, smoking, and alcohol consumption, IL-6 remained significantly associated with reduced progressive motility (β = −0.005) and increased leukocyte concentration (β = 0.0018), and independently predicted bacteriospermia (OR = 1.0325), DFI ≥ 30%, and SDI ≥ 30% (OR = 1.0007 and OR = 1.0006). Although per-unit effect sizes appear modest, the cumulative impact across the IL-6 distribution is clinically relevant and reinforces IL-6 as a dose-dependent indicator of immuno-oxidative stress.

ROC analyses further demonstrated that IL-6 outperformed leukocytes, MDA, and catalase in predicting bacteriospermia and nuclear instability. IL-6 exhibited excellent discriminatory ability for bacteriospermia (AUC = 0.927) and strong predictive performance for DFI (AUC = 0.859) and SDI (AUC = 0.855). These results suggest that a single IL-6 measurement may enhance diagnostic workflows and risk stratification in infertile men.

This study has several limitations. Its cross-sectional design precludes causal inference, and its single-center setting may restrict external validity. Importantly, the study was conceived as an intra-cohort investigation aimed at characterizing internal biological relationships rather than establishing physiological reference values. Determining normative thresholds for IL-6, MDA, catalase, or microbiological parameters will require studies including fertile control groups. Future multicenter investigations involving more diverse populations and integrating additional oxidative markers will be needed to validate and extend these findings. Beyond these aspects, our dataset highlights the promising perspective of developing composite indices integrating IL-6, oxidative markers, and microbial profiles for refined assessment of seminal health.

## 5. Conclusions

This study identifies several novel and clinically relevant insights into seminal inflammation in male infertility. By stratifying IL-6 into threshold-based groups (<30, 30–60, 60–100, and ≥100 pg/mL), we identified distinct and coherent profiles of sperm dysfunction, inflammatory activation, and oxidative imbalance. IL-6 also emerged as an independent predictor of nuclear instability (DFI and SDI ≥ 30%) and bacteriospermia, even after adjustment for age, smoking, and alcohol consumption. Moreover, IL-6 demonstrated superior diagnostic performance compared with leukocyte count, MDA, and catalase for predicting bacteriospermia and nuclear damage.

Taken together, these findings support the potential value of IL-6 as a quantitative biomarker of seminal immuno-oxidative stress and as a candidate tool for refining diagnostic assessment and risk stratification in infertile men. However, these associations remain correlative, and the mechanistic pathways discussed are conceptual rather than experimentally demonstrated within this study.

Future research should include longitudinal and interventional studies, as well as the incorporation of fertile control groups, to establish normative reference values, validate the proposed thresholds, and determine the clinical utility of IL-6 for diagnosis, prognosis, and therapeutic monitoring in male infertility.

## Figures and Tables

**Table 1:** Distribution of age, smoking status, and alcohol consumption across IL-6 groups.

Characteristics	<30	30–60	60–100	≥100	*p*-Value
Age, median (IQR)	35.00 (12)	32.00 (13.5)	39 (15)	31 (10)	0.6891
Smokers, *n* (%)	12 (20.69)	14 (34.88)	3 (7.32)	8 (21.05)	0.1855
Alcohol consumption, *n* (%)	9 (15.52)	7 (16.28)	4 (9.76)	5 (13.16)	0.9376

Note: Age is expressed as median (interquartile range, IQR); categorical variables are presented as absolute counts (*n*) and percentages (%). The Kruskal–Wallis test was used for age comparisons, and the chi-square test for categorical variables.

**Table 2:** Semen, inflammatory, and oxidative parameters by seminal IL-6 concentrations.

	Diagnosis				Statistics	
	<30	30–60	60–100	≥100	Kruskal–Wallis	Dunn’s Post Hoc
Concentration (×10^6^/mL)	35.00 (50.5) ^a^	19.00 (51.5) ^ab^	14.00 (9) ^b^	13.00 (14) ^b^	*p* = 0.0012	<30 vs. 60–100, *p* = 0.0232 <30 vs. ≥100, *p* = 0.0086
Progressive motility (%)	45.40 (23.25) ^a^	30.00 (24) ^ab^	19.00 (26) ^b^	20.00 (20.5) ^b^	*p* < 0.0001	<30 vs. 60–100, *p* < 0.001 <30 vs. ≥100, *p* = 0.0001
Vitality (%)	70.00 (21) ^a^	62.00 (30) ^ab^	45.00 (15) ^b^	53.00 (15) ^b^	*p* = 0.0022	<30 vs. 60–100, *p* = 0.0138 <30 vs. ≥100, *p* = 0.0005
Normal morphology (%)	5.932 (3.745) ^a^	4.705 (0.975) ^a^	4.492 (0.88) ^a^	4.648 (0.80) ^a^	*p* = 0.3781	*p* > 0.05
DFI (%)	13.00 (10.25) ^a^	13.00 (9.5) ^ab^	23.00 (28) ^bc^	37.00 (19.5) ^c^	*p* < 0.0001	<30 vs. 60–100, *p* = 0.037 <30 vs. ≥100, *p* < 0.0001 30–60 vs. ≥100, *p* = 0.0022
SDI (%)	16.00 (9.25) ^a^	16.00 (7.5) ^ab^	27.00 (28) ^b^	34.00 (16) ^b^	*p* < 0.0001	<30 vs. 60–100, *p* = 0.015 <30 vs. ≥100, *p* = 0.0001
Leukocytes (×10^6^/mL)	0.70 (1) ^a^	1.60 (1.85) ^ab^	1.50 (5.4) ^b^	3.85 (3.6) ^b^	*p* < 0.0001	<30 vs. 60–100, *p* = 0.0465 <30 vs. ≥100, *p* < 0.0001 30–60 vs. ≥100, *p* = 0.0224
Bacteriospermia (%)	3.45 ^a^	20.9 ^ab^	39.02 ^b^	76.3 ^c^	*p* < 0.0001	<30 vs. 60–100, *p* = 0.0011 <30 vs. ≥100, *p* < 0.0001 30–60 vs. ≥100, *p* < 0.0001 60–100 vs. ≥100, *p* = 0.0022
MDA (nmol/mg)	423.3 (367.7) ^a^	535.9 (719) ^ab^	670.5 (115.1) ^ab^	964.8 (309.5) ^b^	*p* = 0.018	<30 vs. ≥100, *p* = 0.035
CAT (µmol/min/mg)	467.8 (483.2) ^a^	493.3 (162.3) ^a^	544.9 (625.5) ^a^	572.3 (398) ^a^	*p* = 0.3257	*p* > 0.05

Note: Data are presented as median (interquartile range, IQR), except for bacteriospermia, which is expressed as a percentage (%). Different superscript letters (^a,b,c^) indicate statistically significant differences between IL-6 groups (*p* < 0.05). Groups sharing at least one letter are not significantly different. Abbreviations: DFI, DNA fragmentation index; SDI, sperm chromatin decondensation index; MDA, malondialdehyde; CAT, catalase activity.

**Table 3:** Diagnostic performance of IL-6, leukocyte concentration, MDA, and catalase activity for predicting DFI ≥ 30%, SDI ≥ 30%, and bacteriospermia.

Outcome	Biomarker	AUC	Sensitivity	Specificity	Optimal Cut-Off
DFI ≥ 30%	IL-6	**0.859**	0.727	0.859	68.6 pg/mL
	Leukocytes	0.739	0.773	0.667	1.6 × 10^6^/mL
	MDA	0.684	0.636	0.718	615.453
	CAT	0.617	1.00	0.282	277.569
SDI ≥ 30%	IL-6	**0.855**	0.727	0.859	68.6 pg/mL
	Leukocytes	0.767	0.818	0.679	1.6 × 10^6^/mL
	MDA	0.686	0.636	0.718	615.453
	CAT	0.599	1.00	0.282	277.569
Bacteriospermia	IL-6	**0.927**	0.867	0.871	43.12 pg/mL
	Leukocytes	0.904	1.00	0.714	1.2 × 10^6^/mL
	MDA	0.621	0.40	0.886	801.721
	CAT	0.605	0.967	0.30	277.569

Note: the table reports the area under the curve (AUC), sensitivity, specificity, and optimal cut-off values derived from Youden’s index. Higher AUC values (closer to 1.0) indicate stronger discriminative capacity. Bolded values denote the highest AUCs.

**Table 4:** Multivariate regression analyses examining the independent effect of IL-6 on semen quality parameters (linear regression) and on the probability of abnormal nuclear integrity (DFI ≥ 30% and SDI ≥ 30%) or bacteriospermia (logistic regression).

**Linear Regression**	**β_IL-6 [95% CI]**	**β_Age [95% CI]**	**β_Smoking [95% CI]**	**β_Alcohol [95% CI]**
Concentration	−0.0042 [−0.010–0.001]	−0.0530 [−0.715–0.609]	−17.1088 * [−30.371–−3.846]	3.0560 [−13.237–19.349]
Progressive motility	−0.0050 * [−0.010–−0.000]	0.0264 [−0.500–0.553]	−5.0194 [−15.566–5.527]	3.0089 [−9.947–15.965]
Vitality	−0.0024 [−0.007–0.002]	−0.0974 [−0.606–0.411]	−3.8287 [−14.021–6.364]	6.1703 [−6.351–18.692]
Normal morphology	−0.000035 [−0.000–0.000]	0.0052 [−0.029–0.039]	0.5229 [−0.165–1.210]	−0.5876 [−1.432–0.257]
Leukocytes	0.0018 *** [0.001–0.002]	−0.0041 [−0.053–0.044]	0.6061 [−0.365–1.577]	−0.4327 [−1.625–0.760]
MDA	0.0521 [−0.202–0.307]	15.141 [−14.43–44.71]	642.812 * [50.34–1235.28]	−554.82 [−1282.7–173.02]
CAT	0.0497 [−0.049–0.149]	−2.7222 [−14.225–8.78]	−99.9676 [−330.423–130.5]	4.7130 [−278.4–287.8]
**Logistic regression**	**OR_IL-6 [95% CI]**	**OR_age [95% CI]**	**OR_smoking [95% CI]**	**OR_alcohol [95% CI]**
DFI (≥30%)	1.0007 * [0.00001–0.001]	0.9889 [−0.076–0.054]	1.2835 [−0.991–1.49]	1.0409 [−1.522–1.603]
SDI (≥30%)	1.0006 * [0.00001–0.001]	0.9905 [−0.075–0.056]	2.0203 [−0.504–1.911]	0.8965 [−1.690–1.472]
Bacteriospermia	1.0325 *** [0.018–0.046]	1.0342 [−0.044–0.111]	0.2757 [−3.395–0.819]	0.2645 [−3.807–1.148]

Note: Results are expressed as β coefficients (linear regression) or odds ratios (OR, logistic regression), with 95% confidence intervals (95% CI) [lower–upper]. Asterisks indicate statistical significance (* *p* < 0.05; *** *p* < 0.001).

## Data Availability

The data presented in this study are available on request from the corresponding author. The data are not publicly available due to privacy and ethical restrictions.

## Abbreviations

The following abbreviations are used in this manuscript:

IL-6	Interleukin-6
ROS	Reactive Oxygen Species
MDA	Malondialdehyde
CAT	Catalase
DFI	DNA Fragmentation Index
SDI	Sperm DNA Decondensation Index
OR	Odds Ratio
β	Regression Coefficient (beta coefficient)
*p*	Probability Value (*p*-value)
pg/mL	Picograms per Milliliter
PRR	Pattern Recognition Receptor
MAPK	Mitogen-Activated Protein Kinase
NF-κB	Nuclear Factor kappa-light-chain-enhancer of activated B cells
